# Inhibitory effects of cassiae semen extract on the formation of 2-amino-1-methyl-6-phenylimidazo [4,5-b] pyridine (PhIP) in model system

**DOI:** 10.3389/fnut.2024.1407007

**Published:** 2024-06-05

**Authors:** Di Yu, Youyou Li, Donghua Jiang, Fanlei Kong

**Affiliations:** School of Pharmacy, Guangxi University of Chinese Medicine, Nanning, China

**Keywords:** model system, heterocyclic amine, cassiae semen, byproduct, inhibiting effect

## Abstract

**Introduction:**

2-Amino-1-methyl-6-phenylimidazole [4,5-b] pyridine (PhIP), a heterocyclic amine (HAA), is found in meat products heated at high temperatures. However, PhIP is a mutagenic and potential carcinogenic compound. Cassiae semen, a type of medicine and food homology plant, is abundant in China and has been less applied for inhibiting heterocyclic amines.

**Methods:**

To investigate the inhibitory effect of cassiae semen extract on PhIP formation within a model system and elucidate the inhibitory mechanism, an ultrasonic-assisted method with 70% ethanol was used to obtain cassiae semen extract, which was added to a model system (0.6 mmol of phenylalanine: creatinine, 1:1). PhIP was analyzed by LC–MS to determine inhibitory effect. The byproducts of the system and the mechanism of PhIP inhibition were verified by adding the extract to a model mixture of phenylacetaldehyde, phenylacetaldehyde and creatinine.

**Results:**

The results indicated that PhIP production decreased as the concentration of cassiae semen extract increased, and the highest inhibition rate was 91.9%. Byproduct (E), with a mass–charge ratio of m/z 199.9, was detected in the phenylalanine and creatinine model system but was not detected in the other systems. The cassiae semen extract may have reacted with phenylalanine to produce byproduct (E), which prevented the degradation of phenylalanine by the Strecker reaction to produce phenylacetaldehyde.

**Discussion:**

Cassiae semen extract consumed phenylalanine, which is the precursor for PhIP, thus inhibiting the formation of phenylacetaldehyde and ultimately inhibiting PhIP formation. The main objective of this study was to elucidate the mechanism by which cassiae semen inhibit PhIP formation and establish a theoretical and scientific foundation for practical control measures.

## 1 Introduction

Heterocyclic amines (HAAs) are carcinogenic and mutagenic compounds found in meat products that are cooked at high temperatures. At present, more than 30 different HAAs have been identified and isolated (1). According to formation temperature, HAAs can be classified into thermic HAAs (IQ type) and pyrolytic HAAs (non-IQ type), with the IQ type forming at temperatures between 100 and 300°C and the non-IQ type forming at temperatures greater than 300°C (2). Among the HAAs known to data, PhIP has been established to be the most abundant and most common (3). PhIP, an IQ type HAAs, is a result of dehydration, cyclization and condensation of creatinine and phenylalanine in a system (4). PhIP is a class 2B carcinogen listed by the International Agency for Research on Cancer (IARC) (5). Epidemiologic data indicate that PhIP causes mutations following metabolic activation, which can result in colon, breast, liver, and stomach cancers (6). During the production and processing of meat products, the formation of PhIP is almost inevitable. Effectively controlling the formation of PhIP is highly important for human health and food safety.

A number of studies suggest that PhIP is produced by the reaction of phenylalanine with creatinine. The Strecker degradation reaction transforms phenylalanine to phenylacetaldehyde. Afterward, an aldol condensation reaction with hydrolyzed and cycled creatinine results in the formation of a butyl aldol dehydration product. Finally, PhIP is formed from the aldol condensation product, which undergoes a Schiff base reaction. According to ^13^C labeling, the benzene ring of phenylalanine is a constituent of PhIP, whereas the imidazole ring is derived from creatinine (7). In addition, a recent study revealed that the main thermal degradation product of creatinine was formamide, which reacted with the aldol condensation product to close the pyridine ring, leading to the formation of PhIP, and the reaction also involved active free radicals (8–14). Studies have shown that the addition of fruit extract, rosemary oleoresin or olive oil during beef processing can inhibit PhIP formation (12–14). Quelhas et al. (15) marinated beef in green tea for several hours before frying and found that the content of PhIP decreased significantly compared with that in the blank control group. Wong et al. (16) reported that six vitamins, including riboflavin (VB2), nicotinic acid (VB3), pantothenic acid (VB5), folic acid (VB9), cobalamine (VB12) and pyridoxal hydrochloride (VB6), inhibited PhIP formation by more than 40% in a simulated system and in fried roasted beef. Linghu et al. (17) studied the inhibitory effects of amino acids (tryptophan, lysine, proline, leucine, methionine, valine, threonine, phenylalanine, and aspartic acid) on PhIP. The results showed that PhIP inhibition and the phenylacetaldehyde scavenging activity of amino acids were correlated when phenylacetaldehyde and amino acids were heated. On the one hand, antioxidants are being added (13, 18) to food products to inhibit the formation of HAAs. On the other hand, the precursor of PhIP could also inhibit the formation of HAAs. Yu et al. (19) reported that creatinine inhibited PhIP formation by forming adducts with hydrogen bonds at the N^2^ and N^3^ sites.

Cassiae semen, which belongs to Leguminosae, can be divided into *Cassia obtusifolia L*. and *Cassia tora L.*, and its medicinal parts mainly consist of the dried, mature seeds (20). Cassiae semen, a type of medicine and food homology plant, is abundant in China. This plant contains anthraquinone, naphthopyranone, fatty acids, amino acids, inorganic elements and other chemical components, among which the most important are anthraquinone components (21). The plant exhibit antiaging, anti-inflammatory, free radical scavenging and antibacterial effects (22–27). Extract of cassiae semen was generated in previous studies (28, 29), and it was chemically analyzed and standardized. Studies have shown that the main components of cassiae semen are better extracted through ultrasonic extraction with ethanol as the solvent; compared to traditional methods, this method is less time consuming and generates a greater yield (30).

The ultrasonic-assisted method was used to obtain cassiae semen extract in this work. Based on the existing research, the inhibitory effects of different amounts of cassiae semen extract on the formation of PhIP were investigated in phenylalanine and creatinine (1:1) model systems. To better characterize the probable inhibitory pathway of cassiae semen extract on the formation of PhIP, the effects of cassiae semen extract on precursors of PhIP (phenylalanine/creatinine), intermediates (phenylacetaldehyde), byproduct and PhIP were further evaluated in the model system to clarify the underlying inhibitory pathways. Therefore, the main objective of this study was to elucidate the mechanism by which cassiae semen inhibit PhIP formation and establish a theoretical and scientific foundation for practical control measures, providing valuable information for improving food safety.

## 2 Materials and methods

### 2.1 Materials

All the chemicals and solvents used were of mass spectrometry or analytical grade. PhIP was purchased from Toronto Research Chemicals (North York, Ontario, Canada). Creatinine, L-phenylalanine, and phenylacetaldehyde were purchased from Shanghai McLean Biochemical Technology Co., Ltd. (Shanghai, China). Cassiae semen was purchased from Tongrentang (Bozhou, China). All the other chemicals used were purchased from Semerfer Technology Co., Ltd. (Shanghai, China), Chengdu Cologne Chemicals Co., Ltd. (Chengdu, China), Shanghai Jizhi Biochemical Technology Co., Ltd. (Shanghai, China) or Jiangsu Kaiji Biotechnology Co., Ltd. (Jiangsu, China). All the solutions were prepared with deionized water. Experimental research on plants, including the collection of plant material, was carried out in compliance with institutional, national or international norms and legislation.

### 2.2 Preparation of cassiae semen extract

Based on previous extraction methods (31), cassiae semen extract was prepared by an ultrasonic-assisted method using ethanol as the solvent. A solution of cassiae semen was extracted in an ultrasonicator at 70°C for 1 h before 70% ethanol was added at a material to liquid ratio of 1:40. The mixture was added and centrifuged at 3500 rpm for 10 min. The obtained solution was a concentrated solution, which was frozen in ice cubes in a refrigerator at −18°C. Finally, the solution was evaporated and freeze-dried.

### 2.3 Preparation of the model reaction mixture

The extract (0, 0.01, 0.03, 0.05, 0.10 or 0.15 g) was added to a model system composed of phenylalanine (0.6 mmol) and creatinine (0.6 mmol) and dissolved in 10 mL of water. The reactants were combined in 25 mL PTFE test tubes with a stainless steel exterior liner. The reaction mixture was placed in a forced air oven at 200°C for 3.5 h. After heating, the containers were placed in a cold-water tank, and the water bath was cooled to a normal temperature. The samples were filtered through a 0.22 μm pore filter to generate the sample solution. The samples were stored at 4°C for further analysis.

### 2.4 Preparation of phenylacetaldehyde/creatinine/cassiae semen extract and phenylalanine/cassiae semen extract in the reaction mixture

In order to explore the mechanism by which cassiae semen inhibits PhIP formation, cassiae semen extract (0.05 g) was added to two separate systems composed of phenylacetaldehyde, creatinine (0.6 mmol, 1:1) and phenylalanine (0.6 mmol), and dissolved in 10 mL of water. The reactants were combined in 25 mL PTFE test tubes with a stainless steel exterior liner. The reaction mixture was placed in a forced air oven at 200°C for 3.5 h. After heating, the containers were placed in a cold-water tank, and the water bath was cooled to a normal temperature. The samples were filtered through a 0.22 μm pore filter to generate the sample solution. The samples were stored at 4°C for further analysis.

### 2.5 Determination of the thermal degradation components of cassiae semen extract

First, 0.05 g of cassiae semen extract was weighed accurately and 10 mL of water was added. The reactants were combined in 25 mL PTFE test tubes with a stainless steel exterior liner. The reaction mixture was placed in a forced air oven at 200°C for 3.5 h. After heating, the containers were placed in a cold-water tank, and the water bath was cooled to a normal temperature. The samples were filtered through a 0.22 μm pore filter to generate the sample solution. The samples were stored at 4°C for further analysis.

### 2.6 Preparation of the phenylacetaldehyde derivative

Phenylacetaldehyde cannot be directly detected by LC-MS, whereas can the derivative of phenylacetaldehyde through derivatization reactions. The method used to prepare the phenylacetaldehyde derivative was slightly modified according to the methods of Di et al. (32) Phenylacetaldehyde (1 mL) and o-phenylenediamine (1 mL, 1.00 mg/mL) were mixed and placed in a biochemical incubator at 25°C for 12 h to generate benzimidazole (2-PB). The samples were filtered through a 0.22 μm pore filter membrane to generate a sample solution. The samples were stored at 4°C.

The model system reaction solution (1 mL) and o-phenylenediamine (1 mL, 1.00 mg/mL) were mixed. Sample solutions were obtained according to the above method. The samples were stored at 4°C.

### 2.7 LC-MS analysis

Ten microliters of sample were analyzed by an HPLC-MS/MS system (Waters Company, United States), which consisted of a Waters Quaternary Solvent Manager-R pump, Waters Cortecs C18+ (2.7 μm, 2.1 mm × 100 mm) column, and Waters XDR and Waters 2,998 PDA detectors connected to a triple quadrupole mass spectrometer. A positive electrospray ionization interface was used for sample detection, mobile phase A was composed of methanol, and mobile phase C was composed of water and acetic acid (1,000/1, v/v). The elution procedure was as follows: 5–90% A, 0–2 min; 90–80% A, 2–4 min; 80–70% A, 4–5 min; 70–5% A, 5–6 min; and 5% A, 6–7 min. The mobile phase was subsequently transported in isocratic mode at a speed of 0.2 mL/min. ESI positive ion mode and selected ion monitoring (SIR) or multiple reaction monitoring (MRM) mode were used for mass spectrometry. The MS operating conditions were as follows: capillary voltage, 0.8 kV; capillary temperature, 300°C; desolvation temperature, 550°C; and cone voltage, 30 V. In this study, MS/MS fragmentation at m/z 225 to 210 was characterized as PhIP (33), MS/MS fragmentation at m/z 199 to 104 was characterized as byproduct (E), and the m/z 104 and m/z 78 were secondary fragment ions of the phenylalanine standard.

### 2.8 Data analysis

Statistical analysis of the data was performed using Microsoft Excel version 2016, and all tests were carried out three times. The AVERAGE function was used to calculate the mean peak area, and the STDEV function was used to calculate the standard deviation. The experimental results are expressed as the mean and standard deviation.

## 3 Results

### 3.1 Changes in the phenylalanine, phenylacetaldehyde, creatinine, and PhIP contents in the model reaction

**Figure 1:**
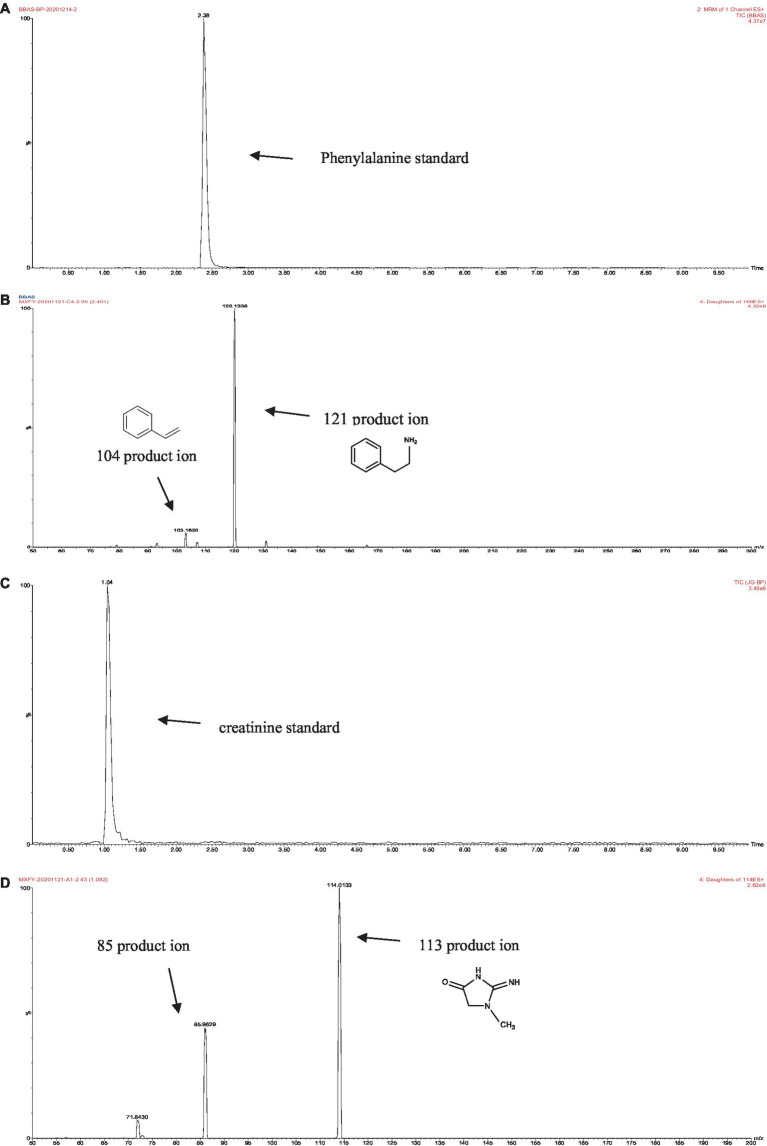
MRM diagram of the phenylalanine standard **(A)**. Secondary fragment diagram of the phenylalanine standard **(B)**. MRM diagram of the creatinine standard **(C)**. Secondary fragment diagram of the creatinine standard **(D)**.

**Figure 2:**
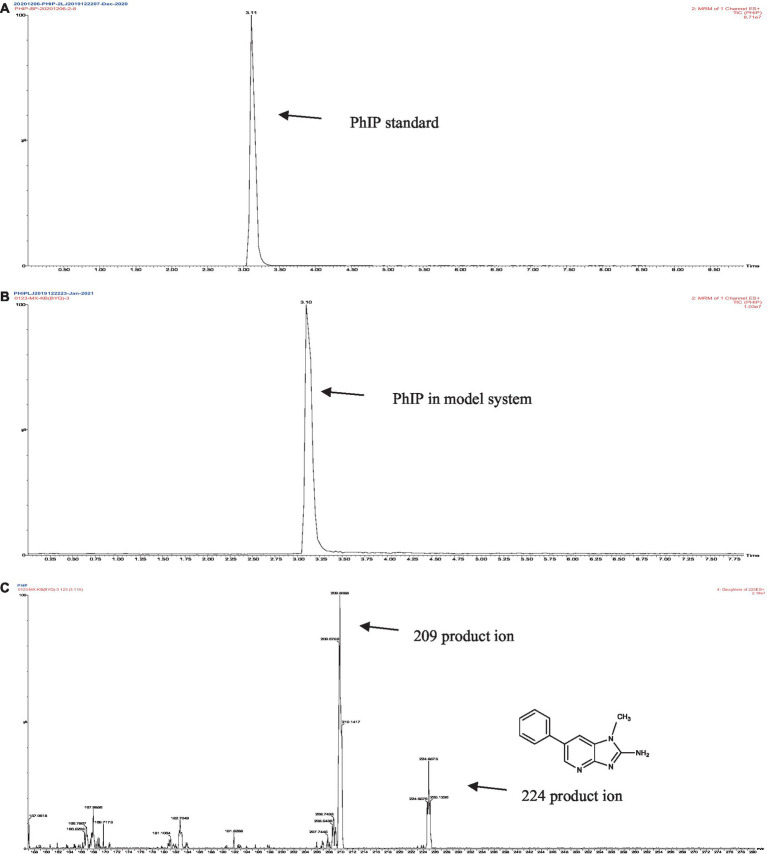
MRM diagram of the PhIP standard **(A)**, MRM diagram of PhIP in the model system **(B)**, Secondary fragment diagram of PhIP in the model system **(C)**.

**Figure 3:**
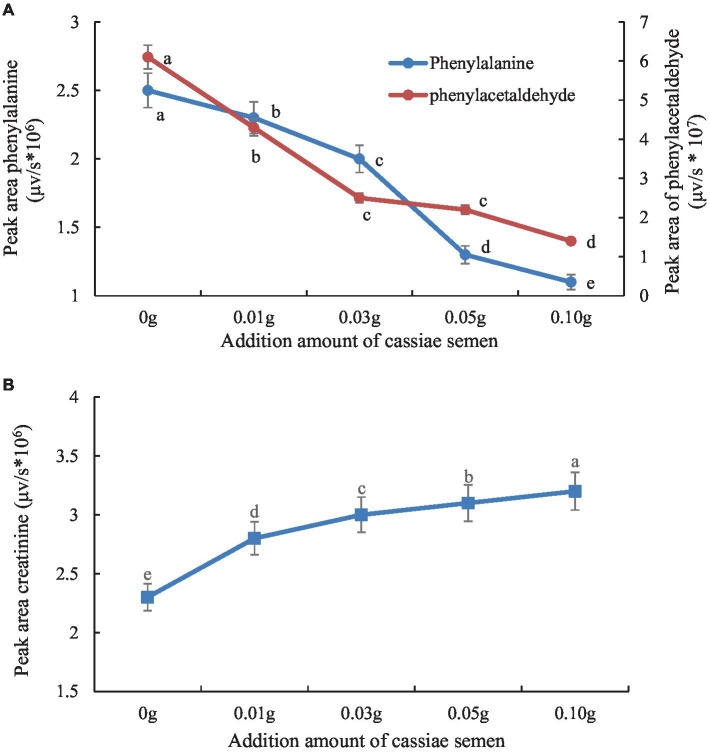
Effect of adding cassiae semen on phenylalanine or phenylacetaldehyde **(A)**, Effect of adding cassiae semen on creatinine **(B)**. (Means with different letters are significantly different *p* < 0.05).

The retention times of the precursor and product were determined based on the standard product. The retention time of phenylalanine was 2.38 min and the main product ions were m/z 104 and m/z 78; the retention time of creatinine was 1.04 min and the main product ion was m/z 86; and the retention time of PhIP was 3.11 min and the main product fragment ion was m/z 209 (Figures 1, 2). The retention time of 2-PB was 3.16 min in MRM mode, and the main product ion was m/z 130 fragment ion. As the amount of cassiae semen extract was increased, the levels of the four compounds changed to different extents; the level of creatinine increased, while the concentrations of the other compounds, phenylalanine, phenylacetaldehyde and PhIP decreased (Figure 3). The inhibitory effect was gradually enhanced with the addition of cassiae semen extract, and the inhibitory rates were 64.7, 68.9, 89.3, and 91.9%, respectively. In the model system, cassiae semen extract strongly inhibited PhIP formation. When the amount of cassiae semen extract was 0.10 g, the rate of ethanol extract inhibition was the greatest, reaching 91.9%. The present analysis revealed that the contents of phenylalanine and phenylacetaldehyde reached a minimum, when PhIP production was minimal, while the content of creatinine reached the maximum. It was speculated that the extract of cassiae semen might consume phenylacetaldehyde or phenylalanine to impede the formation of PhIP, while the consumption of creatinine decreased and its content increased.

### 3.2 Identification of thermal degradation products of cassiae semen extract and byproduct (E)

**Figure 4:**
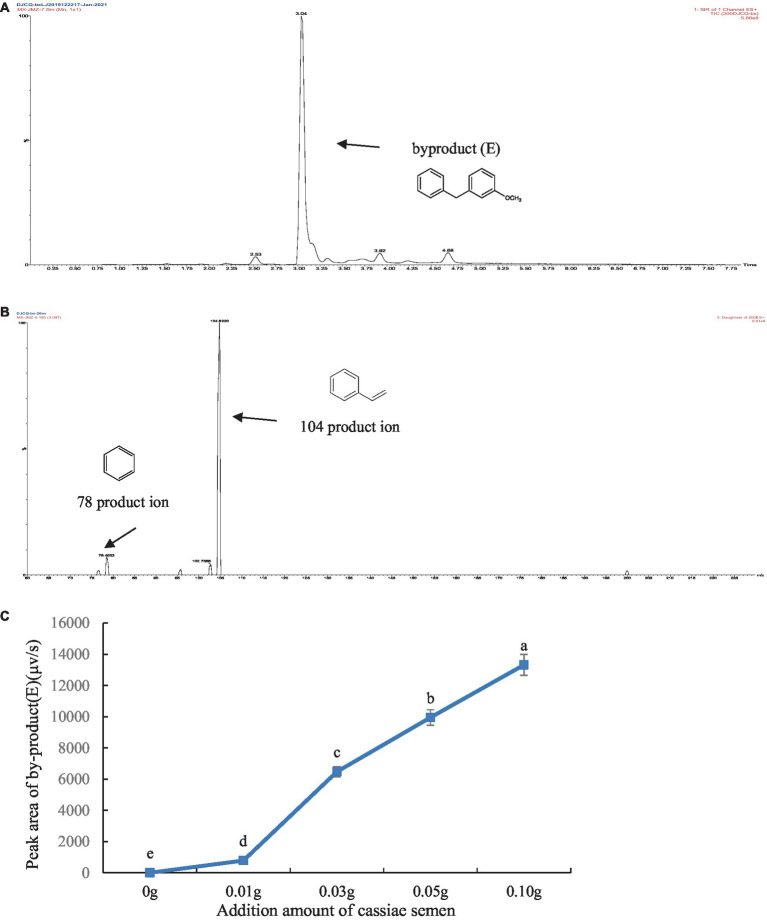
Byproduct (E) SIR scanning pattern diagram **(A)**, Fragment diagram of byproduct (E) **(B)**, Effect of adding cassiae semen on the byproduct (E) **(C)**. (Means with different letters are significantly different *p* < 0.05).

The thermal degradation product of cassiae semen showed a stable peak at 3.09 min, which may have been the main thermal degradation product that reacted with phenylalanine, creatinine or phenylacetaldehyde, thereby inhibiting PhIP formation. In the model systems composed of phenylalanine, creatinine and cassiae semen extract, the [M + H]^+^ peak appeared at 3.04 min with a mass-to-charge ratio of m/z 199.9 in the SIR chromatogram. The secondary mass spectrometry fragments were m/z 104.6 and 78.6 and could be used as a qualitative basis for the generation of the byproduct (E) (Figure 4). To investigate the relationship between the byproduct (E) and PhIP, two systems composed of phenylalanine and phenylacetaldehyde/creatinine were reacted with cassiae semen extract respectively, and the byproducts were monitored.

### 3.3 The relationship between the byproduct (E) and PhIP

**Figure 5:**
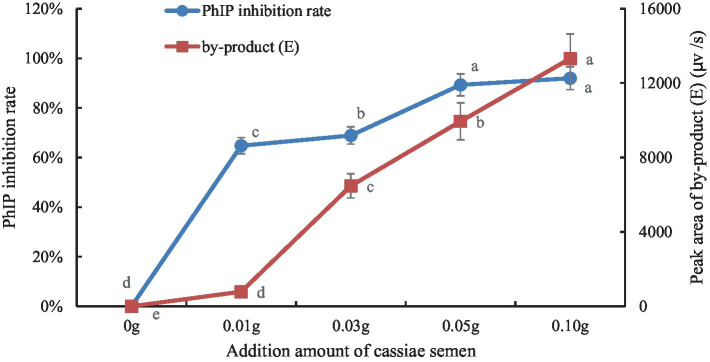
The effect of cassiae semen addition on the inhibition rate of PhIP and the byproducts (E). (Means with different letters are significantly different *p* < 0.05).

To explore the correlation between byproduct (E) and PhIP in model systems, we generated a scatter plot with the amount of cassiae semen as the abscissa and the byproduct content as the ordinate (Figure 4). Moreover, using the correlation function in Excel, the correlation coefficient between the byproduct (E) change and the PhIP inhibition rate was calculated. The variables are more relevant when the correlation coefficient is closer to 1. With an increase in cassiae semen extract, the byproduct (E) concentration increased, and the inhibitory effect of cassiae semen extract on PhIP also increased (Figure 5). The correlation coefficient was 0.8, revealing a strong positive correlation between the change in byproduct (E) and the inhibition rate of PhIP; therefore, the inhibitory effect of the cassiae semen extract on PhIP may be related to byproduct (E). In addition, the byproduct (E) was detected only in the phenylalanine/creatinine and phenylalanine systems and was not present in the phenylacetaldehyde/creatinine system, indicating that phenylalanine, the precursor of PhIP, may be closely related to the byproduct (E) (Table 1).

**Table 1:** System construction and byproduct detection.

System	Phenylalanine	Creatinine	Phenylacetaldehyde	Cassiae semen	Byproduct (E)
1	✓	✓		✓	+
2	✓			✓	+
3		✓	✓	✓	−

### 3.4 Formation of byproduct (E)

In two model systems composed of phenylacetaldehyde/creatinine and phenylalanine, it was found that byproduct (E) could not be detected in the model system of phenylacetaldehyde/creatinine and phenylacetaldehyde. However, the byproduct (E) was present in the model, which contains phenylalanine. The product ion of the byproduct (E) was m/z 104, which was consistent with the product ion of phenylalanine. Moreover, the byproduct (E) content increased as the dose of the cassiae semen extract increased in the phenylalanine and creatinine model system. It was speculated that the formation of byproduct (E) might be related to phenylalanine. Byproduct (E) could not be produced by the reaction of phenylacetaldehyde with cassiae semen extract, but it could be produced by the reaction of phenylalanine with cassiae semen extract, demonstrating that the two substances may be the precursors of byproduct (E).

## 4 Discussion

Many studies have shown that phenylalanine and creatinine are precursors of PhIP. Nevertheless, PhIP is not directly produced by these two precursors, instead, it is created through the transformation of numerous significant intermediates. First, phenylalanine is degraded by the Strecker reaction to form phenylacetaldehyde, which reacts with creatinine to form intermediates after butyraldehyde is produced by aldol condensation. In the subsequent reaction, butyraldehyde dehydration products are produced by dehydration and converted into PhIP. The different reaction stages of PhIP formation involve the phenylalanine, phenylacetaldehyde and butyraldehyde removal, important compounds that form PhIP (1, 7–11, 34).

**Figure 6:**
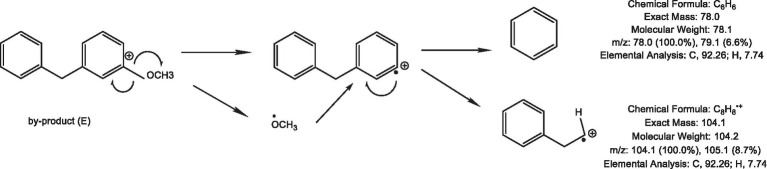
Byproduct (E) fragmentation mechanism.

**Figure 7:**
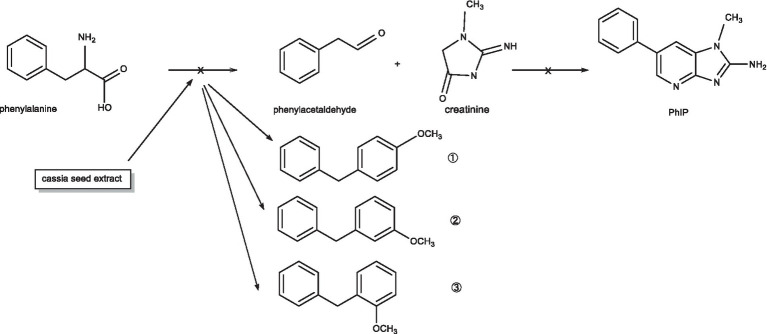
The mechanism by which the extract from cassiae semen inhibits the formation of phenylacetaldehyde.

The experimental results showed that phenylalanine and cassiae semen extract may be precursors of byproduct (E), and the secondary fragment of byproduct (E) at m/z 104.6 may be related to phenylalanine. As shown in Figure 1, the phenylalanine standard ion fragment has a m/z 104. The product ion fragment of the byproduct (E) was broken and rearranged by the parent ion at m/z 199, and the ion fragment at m/z 95 was lost to obtain the compound at m/z 104 (Figure 6). It was speculated that the byproduct (E) was formed by the combination of phenylalanine ion m/z 104 (styrene) and the main thermal degradation product of cassiae semen extract. In addition, the decreases in phenylalanine and phenylacetaldehyde may be related to the reaction between phenylalanine and the cassiae semen extract in the model system, which consumes phenylalanine; as a result, the Strecker degradation of phenylalanine to phenylacetaldehyde is inhibited. Therefore, the inhibitory effect of cassiae semen extract on PhIP may contribute to the inhibition of phenylalanine degradation to phenylacetaldehyde, reducing the content of phenylacetaldehyde and preventing this compound form participatinge in the formation of PhIP (Figure 7). Cassiae semen extract reacts with phenylalanine to produce a byproduct (E). Three different byproduct (E) can be produced, which are denoted as 1, 2, and 3 in Figure 7. As a result of this reaction, some phenylalanine is consumed, which blocks the transformation of phenylalanine to phenylacetaldehyde and alters the 1:1 ratio of phenylalanine to creatinine in the model system. Several studies have shown that creatinine (13), a precursor of PhIP, can also inhibit the formation of excess PhIP. Similarly, in the phenylalanine-creatinine model system with cassiae semen extract, cassiae semen extract inhibited the formation of PhIP through phenylalanine; in addition, excess creatinine remained in the model system, which also inhibited the formation of PhIP. Therefore, combined with previous experimental results, these findings indicate that cassiae semen extract likely inhibits the formation of PhIP by combining with phenylalanine to form a byproduct (E).

## 5 Conclusion

The cassiae semen extract prepared by the ultrasonic-assisted method exhibited an inhibitory effect on PhIP formation in the model system. In the model system composed of phenylalanine and creatinine, as the concentration of cassiae semen extract increased, the levels of phenylalanine, phenylacetaldehyde and PhIP decreased, but the level of creatinine and byproduct (E) increased. With the increase in byproduct (E), the PhIP inhibition rate of the cassiae semen extract also increased. By using correlation function analysis, a strong positive correlation has been found between the change in byproduct (E) concentration and the inhibition rate of PhIP. The inhibitory effect of cassiae semen extract on PhIP may be related to the byproduct (E) with a mass-to-charge ratio of m/z 199.9. The reaction of cassiae semen extract with phenylalanine produces a byproduct (E), which prevents phenylalanine degradation by the Strecker reaction to produce phenylacetaldehyde. Cassiae semen extract consumes phenylalanine, which is the precursor of PhIP, thus inhibiting the formation of phenylacetaldehyde and ultimately inhibiting PhIP formation.

## Data availability statement

The original contributions presented in the study are included in the article/supplementary material, further inquiries can be directed to the corresponding authors.

## Author contributions

DY: Writing – original draft, Writing – review & editing. YL: Writing – review & editing. DJ: Writing – review & editing. FK: Writing – original draft, Writing – review & editing.
